# Governing at the expense of care: diverging growth in administrative and clinical staffing in Swedish health care over 10 years

**DOI:** 10.3389/fpubh.2026.1929890

**Published:** 2026-10-07

**Authors:** Jens Wilkens, Richard Frobell, Björn Ekman

**Affiliations:** 1 Department of Clinical Sciences, Malmö, Lund University, Malmö, Sweden; 2 University College Halmstad and Campus Ängelholm, Halmstad, Sweden

**Keywords:** administrative costs, efficiency, health system governance, staffing, Sweden

## Abstract

Concerns have been raised that Swedish health care resources are increasingly directed toward administrative and managerial functions rather than clinical care. This development may be linked to the growing use of private providers within the publicly funded system. Our study investigates how administrative and managerial staffing has changed over the period between 2014 and 2024 relative to clinical staff categories in Swedish health care. We also investigate to what extent this change can be explained by the increasing use of privately provided care. A purposive database was constructed covering employed staff in governing functions, clinically active staff holding a professional licence or certificate, and regional expenditure on privately provided health care across twenty of Sweden’s regional administrations for the study period (one small region with overlapping municipal responsibilities was excluded). Fixed effects panel regression, controlling for region and year, was used to estimate the association between spending on privately provided care and the number of administrative staff. All staff categories increased in number over the study period, but at markedly different rates: desk-officers grew fastest at 38 percent (27 percent adjusted for population), followed by managers at 28 percent (18), while clinical categories grew more modestly, with physicians increasing by 22 percent (13) and nurses showing the weakest growth at 10 percent (1). Growth in administrative and managerial staff varied considerably across regions but did not show a statistically significant association with regional spending on privately provided care once a common time trend was accounted for. Our findings suggest that Swedish health care has undergone a compositional shift toward administrative roles at a faster pace than clinical roles, a development not attributable to increased private provider use. Future studies should address questions about how administrative growth relates to the value it generates within the health system.

## Introduction

In most countries, there is a general concern that resources are spent on low-value administrative and management functions, generally referred to as ‘bureaucracy’, and that the number of staff working on these tasks is rising over time. A development where administrative functions expand while clinical resources are deprived risks the overall efficiency of the health care system, the trust in a publicly funded health care commitment, and ultimately the trust in the social contract and tax system. At the same time, effective governance of a health care system requires a certain level of administrative capacity. In Sweden, reports documenting the employment development in regional administrations describe how the number of staff in Swedish regions has increased more among non-clinical than clinical staff (1, 2). Related to this concern is the widespread perception among clinical staff that budgets are shrinking, even though overall health care spending has increased continuously. In Sweden, total health spending per capita increased by 12% in real terms between 2014 and 2024 (3).

Over the past two decades, Sweden’s health system has changed in its service delivery structure in several ways, such as the relative shift from in-patient to out-patient care capacity and utilization. Another shift, which may affect the need for administrative capacity, is the move from traditionally integrated organisation of regionally owned, budgeted and managed facilities to more use of private entities. The shift in legal status and ownership of providers started during the 1990s, enabled by reforms to separate purchaser and provider roles and implemented by both traditional procurement mechanisms and enhanced patient choice and enrolment policies, which consequently increased publicly funded private health care provision (4). When more services are purchased on the market, as opposed to provided by budgeted health facilities within the public administration, new administrative roles and capacities are needed. The contractual relationship between the purchaser and provider means the actual assignment to providers needs to be defined and contracts need to be crafted (5). Auditing is given larger prominence to manage the new legal relationship between public purchasers and private providers. Arguably more general accountability concerns are added to governing the system, which requires staff resources in the regional administrations. This is reflected in countries with an insurance-based health care system that tend to have higher administrative costs than countries where funding and provision are integrated, or so called national health systems, also when the comparison is limited to single purchaser systems (6).

This article aims to investigate the volume development in administrative and managerial staff functions in the Swedish health system. Specifically, it aims to describe the change over time as compared to how clinical staff categories have developed, and to estimate to what extent the change in administrative and managerial staff can be explained by the increasing use of private providers.

## Materials and methods

### Data

There is no single data source comprehensively capturing the number of individuals active in clinical work versus central administrative functions that includes all staff regardless of ownership or legal status of providers. The shift in provider structure means that comparisons of staff resources cannot rely on employment data from regional administrations. Thus, we have built a unique purposive database including, by region, employed staff working in governing functions across Sweden’s 21 regional administrations responsible for purchasing health care on behalf of their resident populations (7), clinically active staff holding either a licence or certificate issued by the National board of health and welfare (8), and data on expenditure for health care provided by entities that are not owned by the purchasing region (9). The privately provided health care in Sweden is dominated by for-profit entities but also include relatively small shares of not-for-profit facilities. The data on these purchases also include a small share of spending on public entities outside the purchasing region. A health sector price deflator is used to adjust spending for changes in purchasing power over time. For governance staff, we separate managerial positions and desk-officer roles, such as analysts and administrators. Desk-officer is used as a collective term for the two categories *Handläggare* and *Administratörer* in the Swedish work identification system. Clinically active staff are grouped in three: physicians; nurses (including midwifes); and all other medical staff (e.g., biomedical scientists, physiotherapists and optometrists). As these data on staff are matched with employment status submitted from health care providers to Statistics Sweden, they geographically represent the individual’s workplace and not residency. Table 1 describes the variables from the first year in the material (2014) and the last (2024), corresponding to the time-frame with officially available data for the respective sources. Care for older people and social services under the responsibility of the 290 Swedish municipalities are excluded from the dataset, as well as dentistry. Region Gotland, a small island region with overlapping municipal responsibilities, is also excluded.

**Table 1:** Descriptive statistics of variables across 20 regions in Swedish health care.

Year/Statistics		Population	Medical doctors (end year 2023)	Nurses	Other medical professions (end year 2022)	Managers	Desk- officers	Spending on external providers
*N* = 20 regions		(x1000)	(per 1,000 pop)					SEK (US$) per capita
2014	Mean	484	3.8	10.8	17.7	1.1	2.2	3,280 (338)
	SD	551	0.7	1.1	1.9	0.2	0.3	1,143 (118)
	Min	127	2.9	8.8	14.9	0.8	1.8	1,499 (155)
	Max	2,196	5.4	13.6	22.8	1.6	3.1	6,270 (646)
2024	Mean	526	4.4	11.1	18.9	1.4	3.0	4,675 (482)
	SD	618	0.7	1.1	1.9	0.2	0.5	1,626 (168)
	Min	133	3.6	9.3	16.5	1.0	2.0	1917 (198)
	Max	2,472	5.8	13.7	24.4	1.8	3.8	8,682 (895)

### Statistical methods

To describe the change in staff categories over time, we measure the accumulative changes in employment by staff categories from 2014 to 2024 in crude numbers (adjusted and non-adjusted for population size). In addition, we compare the change across regions adjusted for regional population growth to understand if the changes are uniform or differ geographically. To estimate if the increasing use of private providers can explain the change in administrative staff capacity, we apply a fixed effects panel data regression analysis (Equation 1) including both region and year fixed effects. The former absorbs all time-invariant differences across regions (e.g., large differences in size between regions) and the latter controls for unobserved common temporal trends shared by all regions (e.g., changes in reporting requirements imposed nationwide).


$$\ln(Y_{\mathrm{it}})=\beta \;\ln(X_{\mathrm{it}})+\gamma_{t}+\alpha_{i}+\varepsilon_{\mathrm{it}}$$ (1)

In Equation 1, *Y* is the combined number of administrative and managerial staff employed in regional administrations, *X* is million SEK spent on health care from providers not owned by the region, *i* and *t* denotes region and year, *γ_t_* and α_i_ are fixed effects for year and region, respectively. Clustered standard errors at the region level are used. The estimated *β* is interpreted as the percentage change in the number of staff associated with a percentage change in regional spending on privately provided health care (i.e., the elasticity), within the same region over time, holding constant common year effects. We also test alternative specifications of the model (see sensitivity analysis below).

## Results

The cumulative change in number of staff (Figure 1) shows that all categories rose between 2014 and 2024. However, they grew at substantially different rates, with desk-officers being the fastest growing category. Overall, this professional group increased by 38 percent over the study period (27 percent adjusted for population). The increase in managerial staff was 28 percent (18) across the 20 regional administrations; a similar but less pronounced trend as compared to their subordinate colleagues.

**Figure 1:**
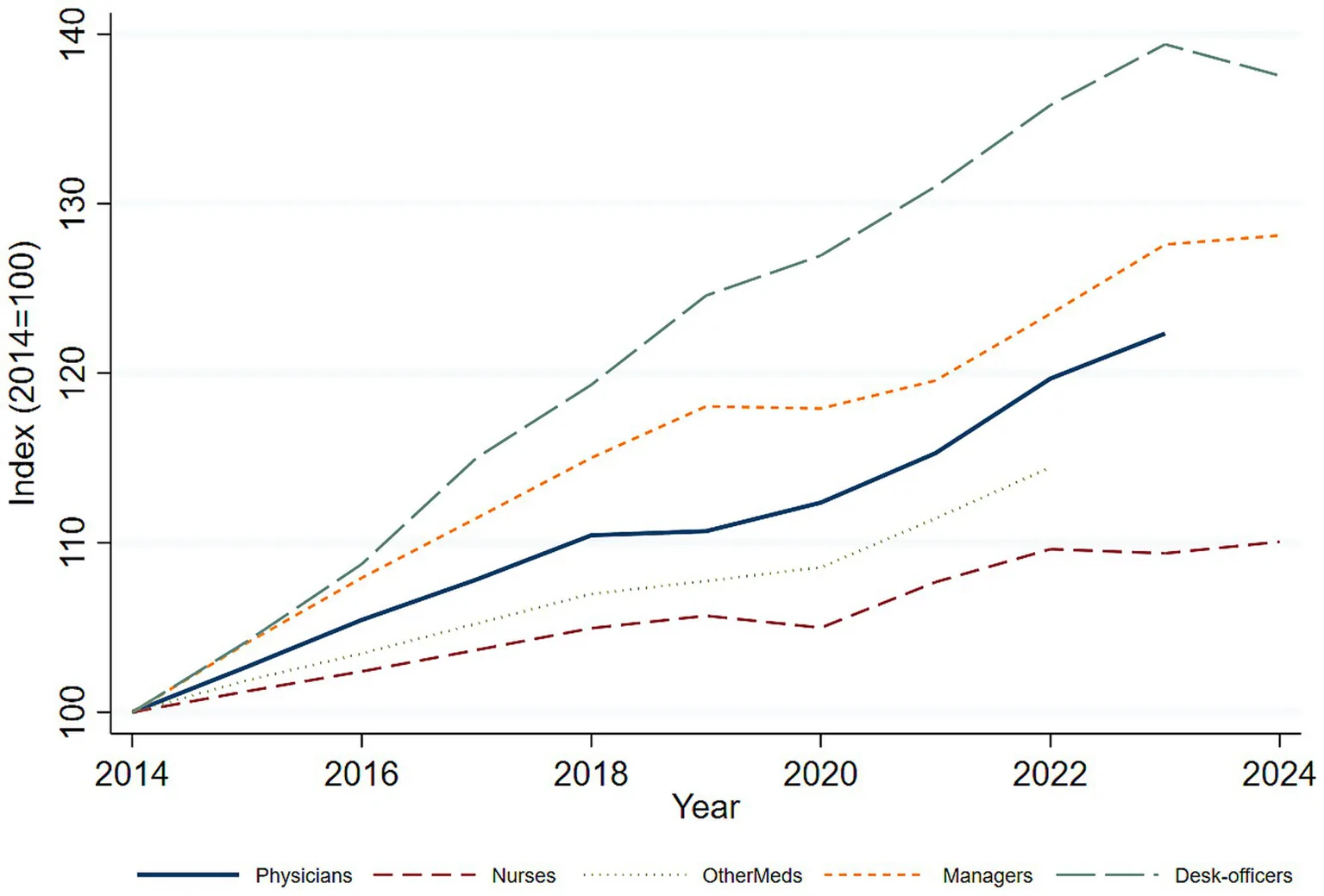
Cumulative change in number of staff for five professional groups.

Among clinical staff, the increase was more modest. The number of physicians rose 22 percent (13 percent adjusted for population) up to 2023, closely followed by the broad category other medical professions, which also displayed a steady upward trajectory. In contrast, nurses exhibited the weakest growth with an overall increase of 10 (1) percent. Consequently, the non-clinical roles have expanded more rapidly than core clinical occupations.

The Swedish health system is characterised by its decentralised governance, with substantial autonomy for regions. The cumulative change by staffing categories showed large variation across the 20 Swedish regions included in the study (Figure 2). The expansion of regional governing staff was not prevalent across all regions. The 2014 to 2024 change in managers varied from −8 to 63 percent whereas the number of desk-officers varied from 7 to 73 percent between regions. The change in the two categories correlated vaguely with a one-unit increase in desk-officers associated with a 0.12 increase in managers (*p* = 0.001).

**Figure 2:**
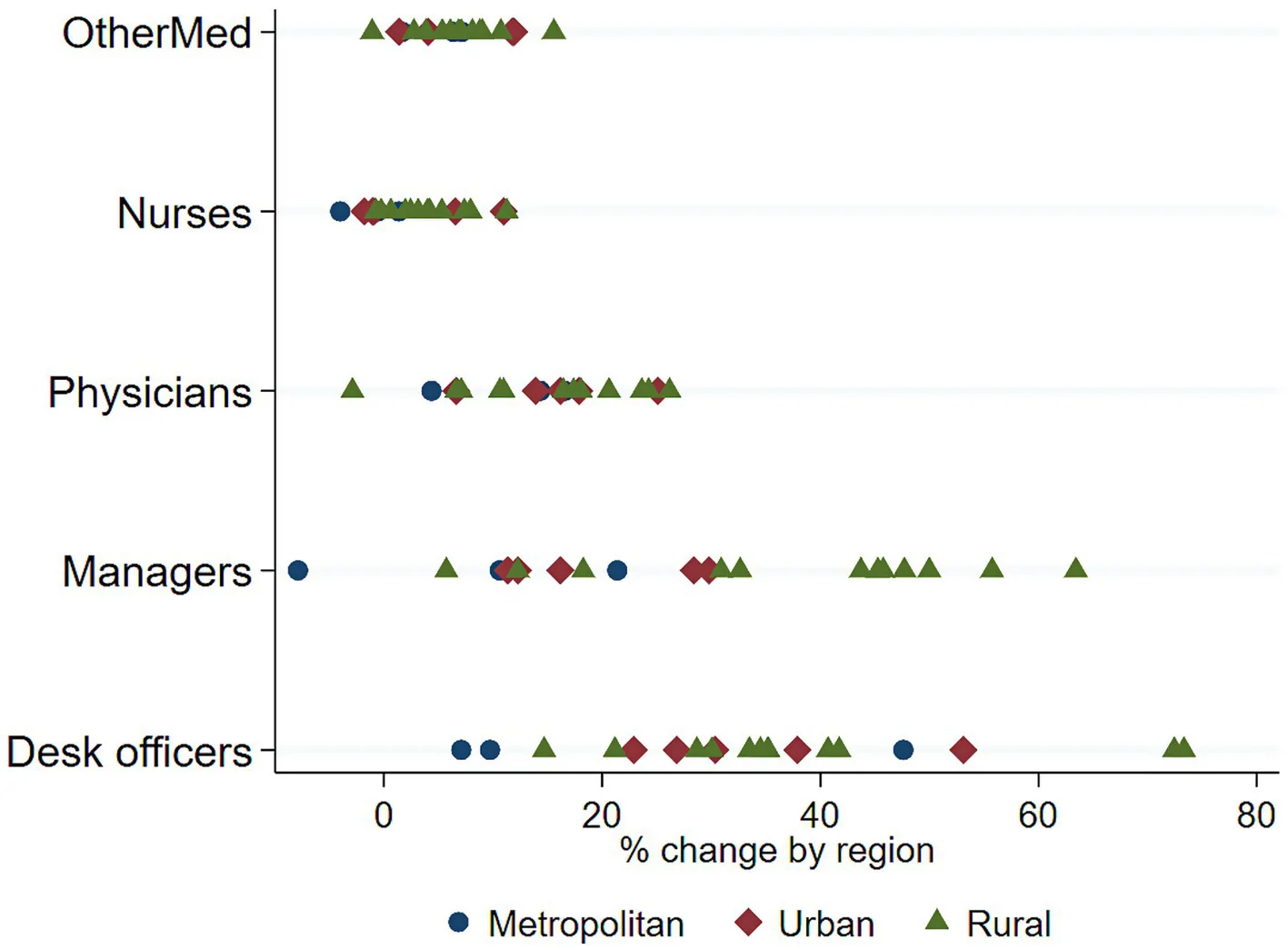
Increase of staff by categories and regions (Metropolitan regions: Stockholm, Västra Götaland, Skåne. Urban regions: Östergötland, Jönköping, Uppsala, Örebro, Halland. Rural regions: Dalarna, Värmland, Gävleborg, Västmanland, Sörmland, Kalmar, Västerbotten, Norrbotten, Blekinge, Jämtland, Kronoberg, Västernorrland).

In the clinical staff categories, all with lower overall growth, the variation between regions was lower. Even though the number of physicians grew nation-wide, the expansion was heterogenous. Growth ranged from negative (−3 percent, adjusted for population growth) to 26 percent (end year 2023). For nurses, the modest increase in staff capacity was relatively evenly distributed, from −4 to 11 percent. In total, seven regions showed negative growth of nurses (adjusted for population development). Across metropolitan, urban, and rural regions there was no systematic pattern, even though single regions saw staff categories grow at noticeably different rates than the national trend. As a formal test of the differences in growth across occupational categories that are illustrated in Figure 2, we estimated a regression of region-level percentage growth rates on an indicator for administrative occupations (managers and desk-officers), including region fixed effects and clustering standard errors by region. Administrative staff exhibited 23.2 percentage points greater growth than clinical occupations (95% CI 16.1–30.3; *p* < 0.001).

The results from estimating whether the change of governing capacity can be explained by the increased use of private providers show that this is not the case. Applying fixed year effects shows a common time trend rather than a correlation with privately purchased health care. The estimated coefficient on lnX is small and statistically insignificant (*β* = 0.041, *p* = 0.561), while all yearly dummies are positive and statistically significant (Supplementary file). Thus, there is a strong common time trend with growing governing staff resources, which is not captured by within-region variation in spending on private health care provision. To illustrate the weak and statistically insignificant relationship, both variables are residualized with respect to region and year fixed effects (Figure 3). The fitted line represents the estimator (β) from the corresponding fixed-effects regression (Equation 1).

**Figure 3:**
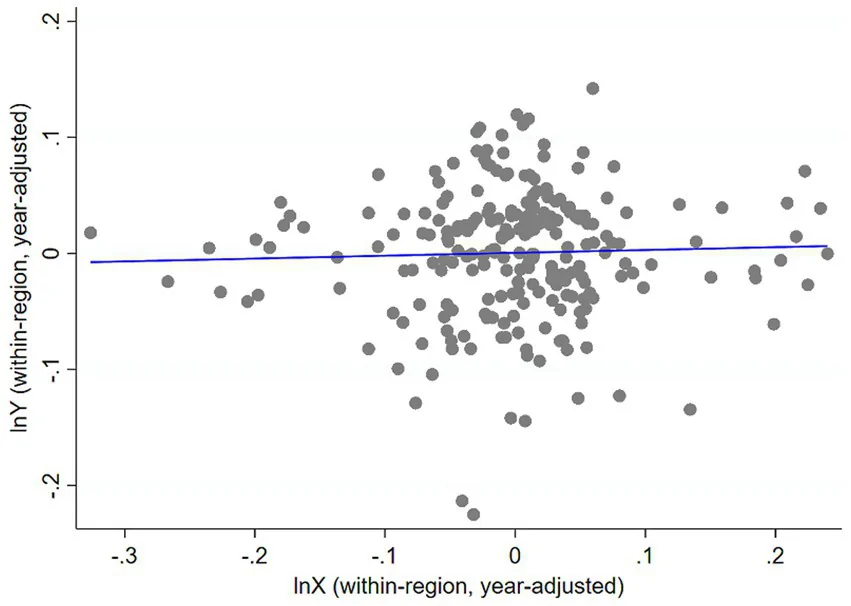
Relationship between spending on private care and governance staff volume.

### Sensitivity analysis

To challenge the model specification, we added a variable for Gross Regional Product (GRP). In Sweden, regional administrations are predominantly funded by a regional labour-income tax. Although there is a national redistribution system in place to equalize differences in ability to collect funds, we wanted to adjust for the possible effect that availability of budget resources in itself fuels growth in administrative capacity. The added regional GRP coefficient was positive (0.340) but not statistically significant (*p* = 0.204). It did not alter the estimated *β* coefficient in the main model. However, it absorbed parts of the variation captured by the year fixed effects suggesting that economic growth and common time trends overlap. In addition, we included two- and three-years time-lag effects (not shown) as well as an aggregation of the data into two-year periods to account for potentially slow-moving adjustments in administrative capacity. None of the time-lagged coefficients displayed statistically significant associations with year fixed effects remaining positive and statistically significant. This supports the results that the observed growth in administrative capacity was driven by common time trends rather than by the increased use of private providers. Since desk-officers increased considerably more than other staff categories, we also tested the inclusion of managers and desk-officers as single dependent variables in separate models. As expected, given the correlation between the two, results remained unchanged.

## Discussion

Our results demonstrate a shift in the composition of the health sector workforce in Sweden, with governing functions increasing most in both absolute and relative size over the study period. We hypothesised that increased purchase of private care can explain an increase, but do not find such relations in this study.

While staff shortages are often reported in Swedish media and by clinicians, we show that the overall number of medical staff in Swedish health care increased over the study period, in line with European trends (10). Notably, the staff categories in this study include many different types of skills and professional roles, between which there can be regional differences in how the number of staff has developed. Indeed, the perceived staff shortages in Sweden are most prominent among certain specialties and geographies, such as physicians and nurses in family medicine and psychiatry, particularly in rural areas (4).

There is no general bench-mark of how much administrative capacity is appropriate or enough in absolute levels in a health system. This study neither relates to the volume of staff at the starting point of the study period, nor attempts to define a potentially appropriate ratio to clinical staff. Instead, we bench-mark the relative volume development of various staff functions over time, and demonstrate that regional health budgets are increasingly spent on governance rather than clinical work. We found that the shift toward increased governing staff capacity was systemic, but also clearly shaped by regional-level choices. Swedish regions vary substantially in size, from 133,000 inhabitants in Blekinge to 2,472,000 inhabitants in Stockholm (excluding the island Gotland). All regions have the same legal framework, similar health care assignments and reporting obligations, meaning a core administrative capacity is probably needed irrespective of population size. At the same time, mere population volume in larger regions probably necessitates relatively more staff.

The study measures growth rates of staff categories; hence we cannot claim causal effects or that one resource substitutes another. Instead, the article’s name “governing at the expense of care” intends to reflect that there are choices to be made in how to allocate resources.

There are also potential alternative explanations for the common time trend, such as the growing data collection for performance monitoring by national health agencies, and data protection and information obligations following the General Data Protection Regulation adopted by the European Union. The steadily increasing digitalization of both clinical management and governance may also drive these trends. These factors may have increased demand for administrative and coordination functions regardless of ownership structure in the health care provision (11). A complementing analytical approach can be to compare the health sector with other industries in society. For example, in the United Kingdom it has been argued that the health sector actually has relatively few managers (12). We recognize that the within-country variation in growth among staff categories is large also in other European countries (13). But workforce consequences of reforms may vary depending on whether reforms are driven primarily through market-oriented governance arrangements such as the Swedish case, or through for example a planned organisational transformation. The opportunity of health care professionals to take part in reform design may also affect how resources are allocated across staff categories as a consequence of reform (14).

Effective governance comes at a cost, which must be part of the overall resource envelope in any health system. Ultimately, any governance capacity, measured in staff or expenditure, should be related to what value it creates. Additional resources for governance are not wasteful if they increase the organisation’s performance in proportion to the additional effort (15). As resources for governance increase, at times with unclear motivation, studies measuring value and results of governing functions in a health system are warranted. Notably, we have found no studies on whether increases in administrative resources of the kind we study translate into improved health outcomes. This aspect arguably receives insufficient attention in health systems assessment and research. The resident population, as the beneficiaries and funders of the system, ultimately expect provision of health care and an equitable distribution of the same in return for their contributions. Studies on the related question about allocating resources specifically to coordination of care, which may be more appealing than general administration, are available. Effect measures are predominantly process indicators rather than outcomes. But even though the evidence is ambiguous, increasing resources for care coordination can lead to improved patient experience of care (16, 17). Clearly, politicians and regional policy makers can increase transparency about how much it costs to administrate health care. Arguably, they should also improve value auditing before allocating additional regional budgets to non-clinical functions.

Further, we acknowledge that data are incomplete in that comparative full-time-equivalents of staff are not available. Neither are data on the use of locum staff, which has increased over time among both public and private providers. However, we have found no indication to how the slower clinical staff increase would have been compensated by more hours by the same people. For locum staff, these individuals are included in our data but by definition float across regions, a movement that is not captured but prominent only in selected smaller regions.

In conclusion, this study presents two main empirical findings from the Swedish context: a structural shift toward administrative functions over clinical roles, and a lack of association with purchasing private care. The study demonstrates a clear general increase in health care staffing across regions in Sweden but with administrative roles as managers and desk-officers displaying the largest increase. We investigated whether increased administration due to governance and purchase of private health care was related to this increase but failed in finding an association.

## Data Availability

The data used in this study are available from the corresponding author upon request.
