# Supplementary material for: Governing at the expense of care: diverging growth in administrative and clinical staffing in Swedish health care over 10 years
Source: Front Public Health. 2026 Oct 7;14:1929890. doi: 10.3389/fpubh.2026.1929890 (PMC13683801; doi:10.3389/fpubh.2026.1929890)
Supplement: Supplementary file 1 [file Supplementary_file_1.pdf]

Annex 1. Fixed-effects panel regression results from main model

Dependent variable: ln Y

Variable	Coefficient	Robust SE	95% CI
ln X	0.041	(0.070)	[-0.105, 0.188]
Year			
2015	0.053***	(0.014)	[0.025, 0.082]
2016	0.084***	(0.017)	[0.048, 0.120]
2017	0.132***	(0.020)	[0.090, 0.174]
2018	0.162***	(0.020)	[0.121, 0.204]
2019	0.219***	(0.026)	[0.164, 0.275]
2020	0.235***	(0.032)	[0.169, 0.301]
2021	0.262***	(0.044)	[0.171, 0.354]
2022	0.302***	(0.044)	[0.209, 0.395]
2023	0.331***	(0.044)	[0.240, 0.423]
2024	0.319***	(0.042)	[0.231, 0.407]
Constant	6.743***	(0.483)	[5.732, 7.753]

Notes: Fixed-effects (within) panel regression; dependent variable is ln Y. Standard errors are clustered by region (20 clusters) and shown in parentheses; 95% confidence intervals are reported in brackets. Year dummies are relative to the omitted base year. *** $p<0.01$, ** $p<0.05$, * $p<0.1$.

Observations = 220; Number of groups (regions) = 20; Obs. per group: min = 11, avg = 11.0, max = 11.

R^2 within = 0.810; R^2 between = 0.770; R^2 overall = 0.169.
